# Ranking-Aware Multiple Instance Learning for Histopathology Slide Classification: Development and Validation Study

**DOI:** 10.2196/84417

**Published:** 2026-02-04

**Authors:** Ho Heon Kim, Gisu Hwang, Won Chan Jeong, Young Sin Ko

**Affiliations:** 1 AI R&D Center, Seegene Medical Foundation Seoul Republic of Korea; 2 Department of Medical Sciences, Graduate School, The Catholic University of Korea Seoul Republic of Korea; 3 Pathology Center, Seegene Medical Foundation Seoul Republic of Korea

**Keywords:** multiple instance learning, learning to rank, digital pathology, whole slide image, weakly supervised learning, mixed supervision, data-efficient training

## Abstract

**Background:**

Multiple instance learning (MIL) is widely used for slide-level classification in digital pathology without requiring expert annotations. However, even partial expert annotations offer valuable supervision; few studies have effectively leveraged this information within MIL frameworks.

**Objective:**

This study aims to develop and evaluate a ranking-aware MIL framework, called rank induction, that effectively incorporates partial expert annotations to improve slide-level classification performance under realistic annotation constraints.

**Methods:**

We developed rank induction, a MIL approach that incorporates expert annotations using a pairwise rank loss inspired by RankNet. The method encourages the model to assign higher attention scores to annotated regions than to unannotated ones, guiding it to focus on diagnostically relevant patches. We evaluated rank induction on 2 public datasets (Camelyon16 and DigestPath2019) and an in-house dataset (Seegene Medical Foundation-stomach; SMF-stomach) and tested its robustness under 3 real-world conditions: low-data regimes, coarse within-slide annotations, and sparse slide-level annotations.

**Results:**

Rank induction outperformed existing methodologies, achieving an area under the receiver operating characteristic curve (AUROC) of 0.839 on Camelyon16, 0.995 on DigestPath2019, and 0.875 on SMF-stomach. It remained robust under low-data conditions, maintaining an AUROC of 0.761 with only 60.2% (130/216) of the training data. When using coarse annotations (with 2240-pixel padding), performance slightly declined to 0.823. Remarkably, annotating just 20% (18/89) of the slides was enough to reach near-saturated performance (AUROC of 0.806, vs 0.839 with full annotations).

**Conclusions:**

Incorporating expert annotations through ranking-based supervision improves MIL-based classification. Rank induction remains robust even with limited, coarse, or sparsely available annotations, demonstrating its practicality in real-world scenarios.

## Introduction

With the rise of digital pathology, whole slide images (WSIs) have become essential for computer-assisted diagnosis tasks such as tumor detection, subtype classification, and prognosis prediction [1-3]. Due to their extremely high resolution, WSIs are typically divided into smaller patches before being used in deep learning models. In fully supervised learning settings, this requires patch-level labels—an annotation process that is time consuming and labor intensive due to the scarcity of lesion-containing patches. To reduce the burden of exhaustive labeling, the multiple instance learning (MIL) framework has emerged as the dominant approach for slide-level prediction. MIL operates in a weakly supervised setting, using only slide-level labels and eliminating the need for detailed patch annotations. Despite this advantage, MIL models still require large datasets to achieve generalizable performance due to a weak supervision signal [4]. In data-constrained settings, MIL training often becomes unstable, leading to a significant reduction in predictive accuracy. To address the limitations of weak supervision, recent studies can be broadly categorized based on the strength of supervision into three main streams: (1) slide-level approaches relying solely on slide labels, (2) pseudolabel-based methods that infer patch-level labels, and (3) expert-guided methods leveraging direct annotations.

Slide-level approaches operate solely with slide-level supervision but aim to improve performance through semisupervised learning, self-supervised learning, or multiresolution architectures [5-12]. For instance, dual-stream multiple instance learning (DS-MIL) introduced a dual-stream architecture that models relationships between high- and low-attention instances to mitigate attention collapse and improve feature discrimination [11]. Hierarchical image pyramid transformer (HIPT) leveraged hierarchical self-supervised vision transformers to capture multiscale contextual information across gigapixel WSIs, demonstrating that strong representation learning can alleviate weak supervision limitations [12]. However, because these approaches still rely only on slide-level labels, the supervision signal remains weak and cannot fully guide the model toward diagnostically relevant regions.

Pseudolabel-based methods generate inferred patch-level labels from attention maps to strengthen the weak supervision signal. A prominent data-efficient training using pseudolabel is clustering-constrained attention multiple instance learning (CLAM), which selects high- and low-attention instances within each slide to form pseudopositive or pseudonegative sets and trains an auxiliary instance classifier [13]. Iterative multiple instance learning proposed an iterative framework that refines both instance and slide representations by retraining the feature extractor with attention-derived pseudolabels, though its performance depends on the stability of the initial MIL attention [14]. In general, pseudolabeling techniques aim to fill the gap in patch-level labels by generating inferred annotations [13-18]. Despite their utility, pseudolabels are inherently noisy, as they are inferred rather than observed. Consequently, MIL pipelines that solely rely on pseudolabels often suffer from degraded performance.

In contrast, expert-guided methods offer a promising way to improve both performance and supervision quality by directly integrating expert annotations. Nevertheless, this approach remains relatively underexplored compared to other categories. A notable study described the attention induction model, which uses pathologist-drawn lesion annotations to guide model attention [19]. However, by enforcing attention scores proportional to the size of the annotated regions, this method inherently biases the model toward larger areas, increasing the risk of overlooking small but clinically critical diagnostic features.

To overcome this limitation, we propose rank induction, a novel MIL framework that introduces a pathology-informed inductive bias. Rather than constraining attention based on region size, rank induction assumes that annotated regions should carry higher diagnostic importance than unannotated ones. By learning relative ranks between patches, our method provides finer-grained supervision, guiding the model to focus on diagnostically relevant areas even when annotations are sparse or training data are limited.

## Methods

### Problem Definition

We extend the standard MIL framework by incorporating patch-level annotations from pathologists as explicit instance labels, either across all training slides or only a subset. In a typical MIL setting, each WSI is divided into *K* patches {x_1_,…,x*_K_*}, and only a slide-level label, indicating disease presence, is provided. A slide is labeled positive if at least 1 patch contains a lesion and negative if all patches are normal (for simplicity, we assume a fixed *K* across slides).

In our extended setting, we additionally use binary labels {y_1_,...,y_k_} at the patch level, where y_k_ ∈{0,1}, y_k_=1, if a patch overlaps with a pathologist-annotated region, and y_k_=0 otherwise. The core inductive bias we introduce is simple but clinically grounded: patches overlapping annotated regions should receive higher attention than unannotated ones. This reflects the assumption that tumor-confirmed regions are more relevant to slide-level diagnosis than those confirmed to be normal.

### Rank Induction

**Figure 1:**
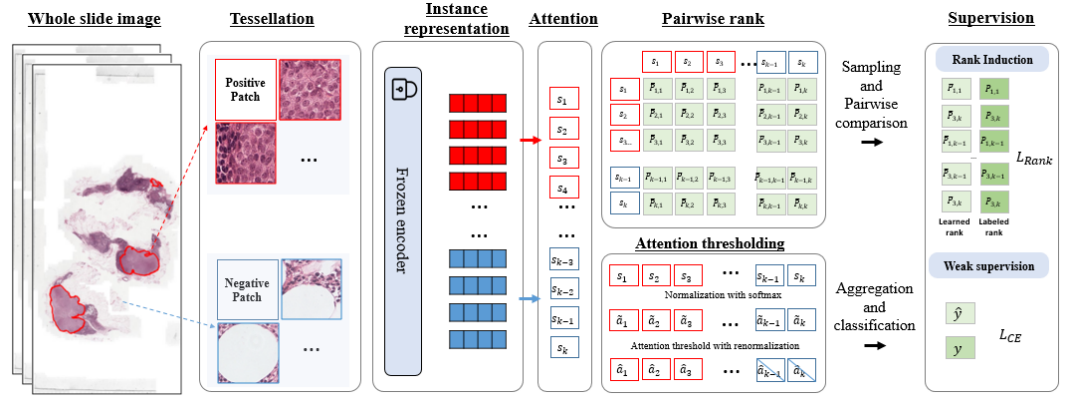
Overview of rank induction.

As illustrated in Figure 1, rank induction consists of 2 key components: pairwise rank comparison derived from rich annotation supervision and attention thresholding to filter out residual noninformative patches. To implement our ranking-aware attention mechanism, we first define the computation of patch-wise attention. Let z_k_ denote the feature vector of patch x_k_ extracted by a backbone feature encoder. We compute a raw attention score s_k_ from z_k_ using an attention layer and normalize it using softmax: 
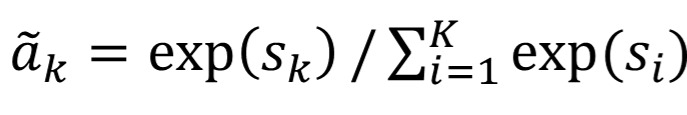
. Inspired by RankNet [20], rank induction introduces a ranking constraint that encourages higher attention scores for annotated patches than for nonannotated ones, such that s_i_ > s_j_, where s_i_ is the attention score for an annotated patch and s_j_ for a nonannotated patch.

To avoid the normalization constraint of softmax (
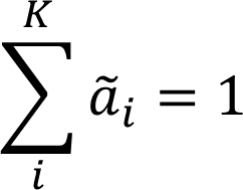
), we apply the ranking loss directly on the raw attention scores s_i_ ε R. We define the pairwise ranking preference using a sigmoid function:


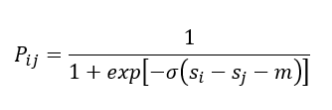


Here, *m* is a margin parameter that enforces separation between positive and negative patches and σ is a scaling factor. We exclude same-class pairs (eg, lesion-lesion or normal-normal) and define the valid pair set: P = {(i,j)|y_i_ = 1, y_j_ = 0}. Let 
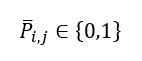
 be the ground truth ranking label. The rank loss is defined as:


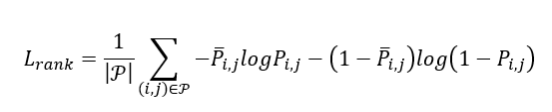


We jointly optimize the slide-level binary cross-entropy loss and the rank loss:

L = L_BCE_ + λL_rank_

where λ is a weighting hyperparameter. For slides without annotated lesions (ie, negative slides), we omit the ranking loss and only apply the slide-level loss.

To mitigate slide-level representation dilution, where a few high-attention lesion patches are overwhelmed by numerous low-attention nonlesion patches during the attention-weighted summation, we apply an attention thresholding strategy during training. Specifically, we threshold the normalized attention weights and then renormalize all (including zero-valued) weights as follows:


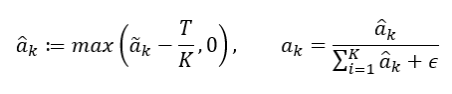


Here, *T* is a predefined threshold and *K* is the number of patches. The threshold *T* is introduced to prevent the slide-level representation from being dominated by the large number of negative instances when the bag size is large. For example, in a slide containing 2000 instances with only 10 lesion patches, even if attention is highly concentrated on those few positives, the aggregate contribution from thousands of near-zero attention weights of negative patches can still overshadow the lesion signal, leading to degraded slide representations. By applying *T*, we effectively suppress these noise-like weights and maintain a more balanced representation across instances.

### Datasets

We evaluated rank induction on 2 public datasets (Camelyon16 [21] and DigestPath2019 [22]) and an in-house dataset (Seegene Medical Foundation-stomach; SMF-stomach). Textbox 1 provides a detailed description of the datasets used in this study, with sample count statistics summarized in Table 1, which presents the statistics for the 3 datasets.

**Description of the datasets used in this study.**

- Camelyon16: this dataset consists of 399 hematoxylin and eosin-stained whole slide images (WSIs) of lymph node sections for breast cancer metastasis detection. It is originally split into 67.7% (270/399) training slides and 32.3% (129/399) test slides. For model development and hyperparameter tuning, we further split the 270 training WSIs into 216 (80%) training slides and 54 (20%) validation slides (an 8:2 split). Each slide includes a slide-level diagnosis and polygon-based lesion annotations provided by expert pathologists. In this study, slides containing metastasis were labeled as abnormal, while negative slides were labeled as normal.
- DigestPath2019: we used the colonoscopy tissue segmentation dataset from the DigestPath Challenge 2019. The dataset originally includes 660 training images and 212 test images with slide-level labels (benign or malignant). However, because the test set is not publicly available, we used only the 660 labeled WSIs. We allocated 20% (132/660) of the slides as the test set and used the remaining 80% (528/660) for training and validation. Specifically, we split the 528 slides into 422 (79.9%) for training and 106 (20.1%) for validation. Each WSI is a JPEG image with an average resolution of approximately 5000×5000 pixels. For consistency, we mapped malignant samples to the abnormal class and benign samples to the normal class.
- SMF-stomach (in-house): we constructed an in-house gastric pathology dataset at Seegene Medical Foundation (SMF). Slide preparation and digitization followed standard operating procedures described by Kim et al [23]. A distinct feature of this dataset, as illustrated in Multimedia Appendix 1, is that at least 3 consecutive tissue sections are placed on a single glass slide; however, pixel-level annotations were not exhaustively applied to every section. A single board-certified pathologist reviewed the slides and provided polygon-based annotations only for representative regions; thus, unannotated tissue sections may coexist within the same WSI. Following the diagnostic criteria of Ko et al [24], lesions were originally categorized as malignant, dysplasia, negative, and uncategorized. We excluded the uncategorized class due to its high heterogeneity. For the binary classification task, we mapped malignant and dysplasia to the abnormal class and negative to the normal class. The final dataset comprised 209 slides, allocated as 120 (57.4%) for training, 30 (14.4%) for validation, and 59 (28.2%) for testing.

**Table 1:** Dataset statistics (whole slide images).

Dataset	Train		Validation		Test	
	Abnormal	Normal	Abnormal	Normal	Abnormal	Normal
Camelyon16	89	127	22	32	49	80
DigestPath2019	160	262	40	66	50	82
SMF-stomach^a^	81	39	20	10	39	20

^a^SMF-stomach: Seegene Medical Foundation-stomach.

### Data Preprocessing

We applied the same preprocessing pipeline to all datasets. First, we performed tissue-background segmentation using the multilevel Otsu algorithm. Each WSI was converted to grayscale and thresholded to generate a binary mask that separated tissue from the white background. We retained only the foreground tissue regions and discarded blank or irrelevant areas.

Next, we tessellated each foreground region into nonoverlapping patches of size 224×224 pixels. For Camelyon16 and SMF-stomach, we extracted patches at 20× magnification, whereas for DigestPath2019, we used the original image resolution.

After tessellation, we computed the tissue coverage ratio for each patch and discarded patches containing less than 5% tissue, as these were considered mostly empty. Finally, we used a ResNet-50 [24] pretrained on ImageNet-1k [25] to extract features from each patch. Specifically, we used the output of the third residual block and applied an adaptive average pooling layer to obtain a 2048-dimensional feature vector.

### Statistical Methods

To evaluate rank induction, we designed a series of experiments targeting performance, data efficiency, and annotation use. Our evaluation included 6 different experiment types, which are discussed in the following subsections.

#### Model Comparison

We trained and evaluated 7 different MIL models on 3 datasets: the baseline attention-based MIL (AB-MIL), the attention induction method, 2 variants of CLAM (single-branch [SB]; multibranch [MB]), DS-MIL, HIPT, and the proposed rank induction method.

#### Low-Data Regime

To test data efficiency, we trained models on subsets of the training data at 20%, 40%, 60%, 80%, and 100% of the full set. For each fraction, we trained all 7 models (AB-MIL, attention induction, CLAM-SB, CLAM-MB, DS-MIL, HIPT, and rank induction) from scratch and evaluated them on a common held-out test set.

#### Annotation Granularity

To simulate real-world coarse annotations, we degraded annotation precision by expanding the original lesion masks on Camelyon16 slides. At 40× magnification, we added symmetric paddings of 0, 448, 896, 1344, 1792, and 2240 pixels to each polygon annotation. For instance, a 448-pixel padding at 40× resolution adds 1 patch-width around the lesion, which corresponds to a 224-pixel patch at 20× magnification—potentially including normal tissue and simulating noisy annotations.

#### Subset-Annotated Slide Setting

In real-world clinical settings, not all slides can be exhaustively annotated. We tested rank induction under partial supervision by varying the proportion of positive slides with patch-level annotations (5%, 10%, 20%, 40%, 60%, and 80%) in the Camelyon16 training set (111 positive slides in total). The remaining positive slides were treated with slide-level labels only. Negative slides, by definition, had no lesion annotations.

#### Ablation Test

To assess the contribution of attention thresholding, we performed an ablation study with threshold values *T*∈{0,0.25,0.5,1,2,5}. The case of *T*=0 served as the reference condition (ie, without attention thresholding). We compared WSI classification performance and the cumulative attention weight assigned to nonlesion patches on the test set to examine how different thresholds affect model behavior.

#### Attention Localization

To assess interpretability, we compared how different MIL models spatially focus on tumor regions in Camelyon16. This is not a dense pixel-wise segmentation map; rather, it is a ranking over discrete patches, with scores defined for all patches in the slide [13]. Because no established gold standard protocol exists for evaluating such attention maps, we quantified spatial alignment between attention and expert lesion annotations using 3 metrics: intersection over union (IoU), Dice score, and pointing game [25]. For each WSI, we ranked all patches by attention weight and then selected only the top α% relative to the number of tumor-annotated patches in that slide (α∈{1, 5, 10, 20, 50, 75, 100}). To ensure that the ideal case could always achieve a score of 1, IoU and Dice score were computed by sampling up to the number of tumor-annotated patches, whereas the pointing game was evaluated by sampling from all patches in the slide. We then computed IoU and Dice score between the selected high-attention patches and the tumor-annotated patches, averaged over positive test slides. We also measured the pointing game by calculating a hit rate, defined as whether at least 1 of the top α highest attention patches fell within the annotated tumor region (classified as a hit). For all malignant test slides, localization accuracy was defined as hits divided by the sum of hits and misses.

For model evaluation, we computed area under the receiver operating characteristic curve (AUROC), area under the precision-recall curve (AUPRC), and accuracy for slide-level classification. All results were averaged over a 10-fold Monte Carlo cross-validation. We assessed statistical significance using the Mann-Whitney *U* test, with *P* values <.05 considered statistically significant.

### Experiment Details

We implemented the models in Python using the PyTorch deep learning framework. WSI data preprocessing was carried out with OpenSlide, OpenCV, and Pillow libraries. Visualization of attention maps and result plots was generated using Seaborn and Matplotlib libraries (specific version information for each library can be found in the GitHub repository). The training and experiments were run on a computing system with a pair of NVIDIA A100 graphics processing units (80 GB memory each) and an Intel Xeon Gold 6338N central processing unit (128 cores, 2.20 GHz) with 512 GB of RAM. Additionally, because the original implementation of attention induction was not publicly available, we reimplemented the method based on the descriptions provided in the paper. Our implementation is available to facilitate reproducibility [26].

For optimization, we used the Adam optimizer with a learning rate of 2×10^–4^ and a weight decay of 1×10^–5^. We trained each model for up to 200 epochs using an early stopping criterion: if the validation loss did not improve for 7 consecutive epochs (after an initial 20-epoch warmup period), training was stopped. In practice, most models converged well before 200 epochs under this criterion. For all MIL benchmarks, the ResNet-50 backbone was frozen during training. Crucially, we used a frozen ResNet-50 backbone for all MIL benchmarks. Although methods such as DS-MIL and HIPT typically leverage self-supervised pretraining or hierarchical encoders, we used ResNet-50 as the feature extractor for this experiment. This ensured a direct comparison of the MIL aggregation strategies themselves, isolating their performance from the variations in pretrained feature representations.

We set the rank loss hyperparameters as follows: the scaling factor σ=1 and margin m=1 in the pairwise probability formula, and the rank loss weight λ=1. In each training iteration, to limit memory use and ensure computational efficiency when computing the rank loss, we randomly sampled up to 1024 positive and 1024 negative patches per slide. The attention threshold *T* was set to 1.

### Ethical Considerations

This study used both publicly available deidentified datasets (Camelyon16 and DigestPath2019) and a private clinical dataset (SMF-stomach). The Camelyon16 and DigestPath2019 datasets consist of fully deidentified WSIs, publicly released for research under their respective challenge licenses; therefore, no institutional review board (IRB) approval or informed consent was required for their use. For the SMF-stomach dataset, this study was approved by the IRB of the Seegene Medical Foundation (SMF-IRB-2024-015). Given the anonymous and deidentified nature of the retrospective pathological images, informed consent was not required.

## Results

### Model Performance

We compared rank induction against 6 representative MIL benchmarks: AB-MIL, attention induction, CLAM, DS-MIL, and HIPT, regarding slide-level classification performance. On Camelyon16, rank induction achieved an AUROC of 0.839 (SD 0.050) and an AUPRC of 0.850 (SD 0.042), outperforming all baseline methods with consistently low variance (Table 2). We observed similar performance trends on the DigestPath2019 dataset. Although all models performed well due to the dataset’s relative simplicity, rank induction achieved the best results (AUROC=0.995; AUPRC=0.993) with stable variance. On the SMF-stomach dataset, rank induction demonstrated the best performance, achieving an AUROC of 0.875 (SD 0.008) and an AUPRC of 0.937 (SD 0.006). Due to space constraints, the full accuracy comparison for all datasets is reported in Multimedia Appendix 2.

**Table 2:** Comparison of model performance across methods on 2 public datasets and an in-house dataset.

Model	Camelyon16		DigestPath2019			SMF^a^-stomach	
	AUROC^b^, mean (SD)	AUPRC^c^, mean (SD)	AUROC, mean (SD)	AUPRC, mean (SD)	AUROC, mean (SD)		AUPRC, mean (SD)
AB-MIL^d^	0.741 (0.146)	0.730 (0.183)	0.993 (0.003)	0.990 (0.005)	0.864 (0.031)		0.928 (0.016)
Attention induction	0.742 (0.142)	0.727 (0.179)	0.994 (0.002)	0.990 (0.003)	0.868 (0.031)^e^		0.930 (0.016)^e^
CLAM-SB^f^	0.732 (0.137)	0.700 (0.179)	0.977 (0.019)	0.965 (0.026)	0.837 (0.023)		0.919 (0.013)
CLAM-MB^g^	0.794 (0.128)^e^	0.767 (0.168)^e^	0.976 (0.019)	0.963 (0.026)	0.838 (0.032)		0.916 (0.025)
DS-MIL^h^	0.690 (0.170)	0.666 (0.223)	0.995 (0.004)^e^	0.993 (0.006)^e^	0.839 (0.031)		0.915 (0.016)
HIPT^i^	0.483 (0.103)	0.416 (0.136)	0.962 (0.058)	0.947 (0.091)	0.676 (0.064)		0.751 (0.060)
Rank induction	*0.839* *(* *0.* *050* *)* ^j,k,^ ^l,m,^ ^o^ ^,p^	*0.850* *(* *0.* *042* *)* ^j,k,^ ^l,m,^ ^o^ ^,p^	*0.995* *(* *0.* *002* *)* ^j,k,^ ^l,m,n,p^	*0.993* *(* *0.* *002* *)* ^j,k,^ ^l,m,n,^ ^p^	*0.875* *(* *0.* *008* *)* ^j,m,^ ^n,o,p^		*0.937* *(* *0.* *006* *)* ^j,m,^ ^n,o,p^

^a^SMF: Seegene Medical Foundation.

^b^AUROC: area under the receiver operating characteristic curve.

^c^AUPRC: area under the precision-recall curve.

^d^AB-MIL: attention-based multiple instance learning.

^e^Second-best result.

^f^CLAM-SB: clustering-constrained attention multiple instance learning, single-branch.

^g^CLAM-MB: clustering-constrained attention multiple instance learning, multibranch.

^h^DS-MIL: dual-stream multiple instance learning.

^i^HIPT: hierarchical image pyramid transformer.

^j^Italicization indicates the best result.

^k^*P*<.05 vs attention-based multiple instance learning.

^l^*P*<.05 vs attention induction.

^m^*P*<.05 vs clustering-constrained attention multiple instance learning, single-branch.

^n^*P*<.05 vs clustering-constrained attention multiple instance learning, multibranch.

^o^*P*<.05 vs dual-stream multiple instance learning.

^p^*P*<.05 vs hierarchical image pyramid transformer.

### Data-Efficient Learning With Expert Annotation

**Figure 2:**
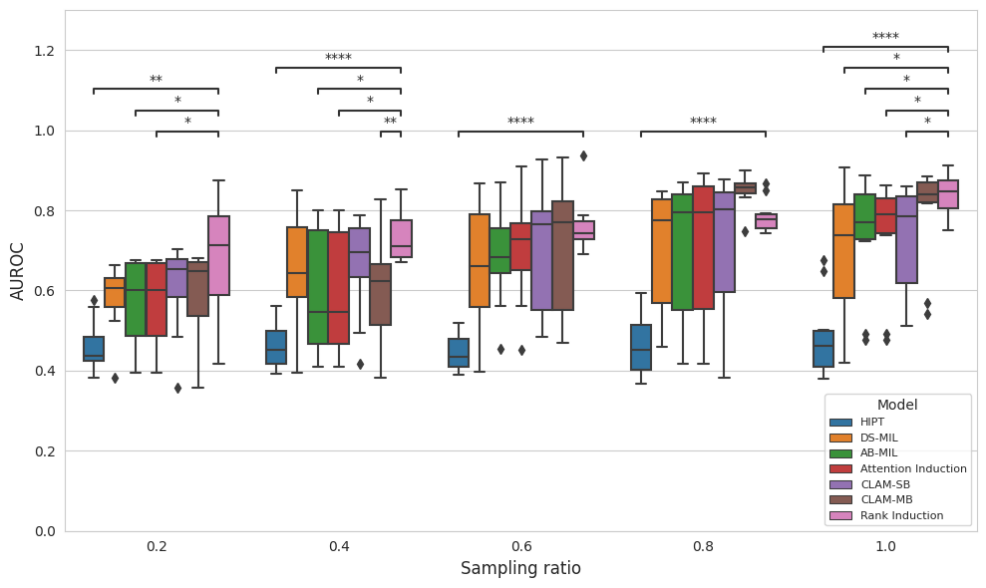
Area under the receiver operating characteristic curve (AUROC) performance across different training set sampling ratios on Camelyon16. AB-MIL: attention-based multiple instance learning; DS-MIL: dual-stream multiple instance learning; CLAM-MB: clustering-constrained attention multiple instance learning, multibranch; CLAM-SB: clustering-constrained attention multiple instance learning, single-branch; HIPT: hierarchical image pyramid transformer. Asterisks indicate statistical significance based on the Mann-Whitney U test: *P<.05, **P<.001, ***P<.0001, and ****P<.00001.

To investigate data-efficient training with expert annotation under low-data regimes reflecting real-world constraints, we assessed model performance on Camelyon16 by varying the proportion of training data (Figure 2). With only 20% of the slides, all methods achieved AUROC scores in the range of 0.4 to 0.6, and statistically significant differences were observed between rank induction and the other benchmarks. Among them, rank induction achieved the highest AUROC. At 40% and 60% of the training data size, rank induction significantly outperformed other MIL methods. When trained with 80% of the data, rank induction achieved an AUROC of 0.786 (SD 0.042), surpassing HIPT (AUROC=0.464, SD 0.076), DS-MIL (AUROC=0.709, SD 0.154), AB-MIL (AUROC=0.707, SD 0.186), attention induction (AUROC=0.714, SD 0.193), CLAM-SB (AUROC=0.721, SD 0.180), and CLAM-MB (AUROC=0.847, SD 0.042).

### Annotation Granularity

**Figure 3:**
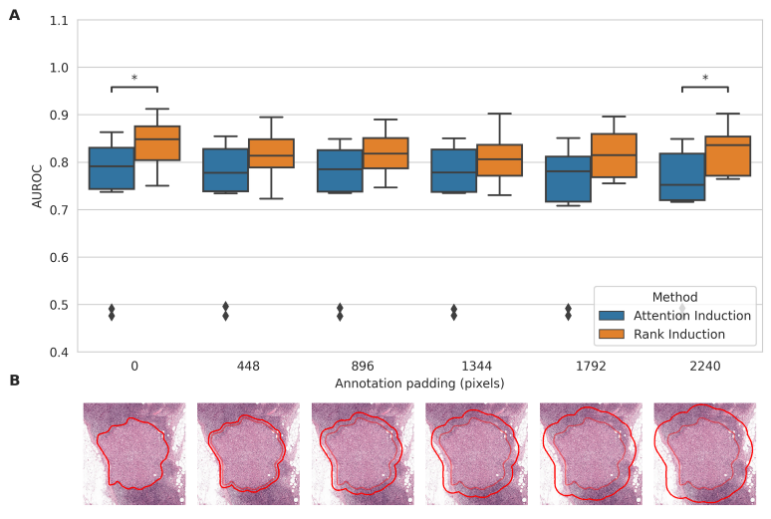
Annotation granularity experiment on Camelyon16. (A) Comparison of slide-level classification performance (area under the receiver operating characteristic curve [AUROC]) between attention induction and rank induction. (B) Representative visualization of the artificially expanded annotations used in the experiment. The thin inner red line indicates the original precise lesion annotation provided by the pathologist (ground truth), while the bold outer red line represents the artificially expanded annotation boundary simulating coarser supervision granularity.

We evaluated model robustness to noisy or imprecise annotations by progressively padding the pathologist-drawn tumor regions on Camelyon16 to simulate coarse lesion markings. Both rank induction and attention induction were trained under each noise condition. With the original annotations (0-pixel padding), rank induction achieved an AUROC of 0.839 (SD 0.050). Even at the highest padding level, its performance declined only slightly to an AUROC of 0.823 (SD 0.050; Figure 3). In comparison, attention induction dropped from an AUROC of 0.742 (SD 0.142) to 0.723 (SD 0.134) as annotation noise increased. Across all levels of annotation coarseness, rank induction consistently outperformed attention induction, although the differences were not statistically significant at some padding levels.

### Fraction of Expert Annotation

**Figure 4:**
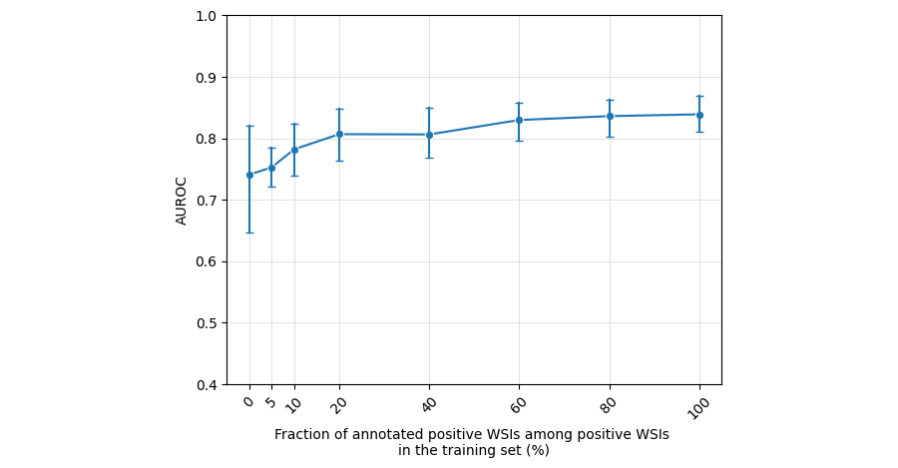
Impact of annotation fraction on area under the receiver operating characteristic curve (AUROC). WSI: whole slide image.

To simulate real-world scenarios in which annotating all WSIs is infeasible, we evaluated model performance by varying the proportion of annotated positive WSIs in the training set. As a baseline (0% annotation), AB-MIL achieved an average AUROC of 0.741 (SD 0.146; Figure 4). With only 10% (9/89) of positive WSIs annotated in the train dataset, rank induction boosted the performance to an average AUROC of 0.782 (SD 0.070). Increasing the annotation coverage to 20% (18/89) raised the AUROC to 0.807 (SD 0.070), while using all available positive WSIs (N=89) yielded a final AUROC of 0.839 (SD 0.050). Notably, rank induction achieved near-saturated performance with 20% of the annotated data, demonstrating high data efficiency. Moreover, the introduction of even minimal annotation significantly reduced performance variance compared to the unannotated baseline. The performance curve began to plateau beyond approximately 20% annotation coverage, indicating diminishing returns in AUROC gains, while maintaining low variability.

### Ablation Test

The impact of varying the attention threshold *T* on model performance and attention distribution is presented in Table 3. As *T* increased from 0 to 2, both AUROC and AUPRC improved steadily, accompanied by a marked reduction in the average sum of attention assigned to nonlesion patches.

**Table 3:** Effect of attention threshold *T* on whole slide image (WSI) classification performance and sum of attention weight for nonlesion patches.

Threshold	Test dataset (n=129)		Positive WSI in the test dataset (n=49; sum of nonlesion attention), mean (SD)
	AUROC^a^	AUPRC^b^	
0	0.830	0.846	0.465 (0.420)
0.25	0.853	0.878	0.411 (0.434)
0.5	0.869	0.881	0.362 (0.423)
1	0.894	0.905	0.269 (0.387)
2	0.916	0.922	0.178 (0.354)
5	0.909	0.914	0.105 (0.339)

^a^AUROC: area under the receiver operating characteristic curve.

^b^AUPRC: area under the precision-recall curve.

With no thresholding (*T*=0) or low thresholds (*T*<1, where attention distributions remain nearly uniform), the AUROC remained relatively low. This suggests that weakly attended nonlesion patches diluted the slide-level representation. The best performance was observed at *T*=2 (AUROC=0.916; AUPRC=0.922), indicating that moderate thresholding effectively suppresses noise-like, low-value attention weights while preserving discriminative focus. Excessively high thresholds (eg, *T*=5) yielded a slight decline, reflecting an optimal balance near *T*=2.

### Attention Localization and Qualitative Analysis

**Figure 5:**
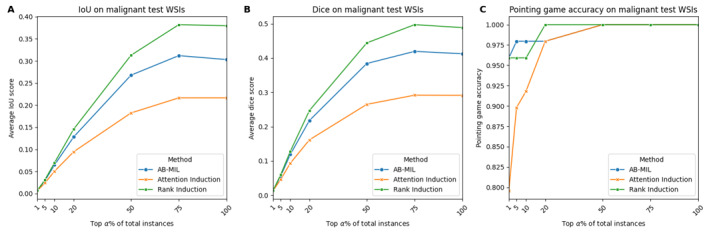
Attention localization performance of attention-based multiple instance learning (AB-MIL), attention induction, and rank induction. (A) Intersection over union (IoU) score according to the top α% of patches. (B) Dice score according to the top α% of patches. (C) Average pointing game score. WSI: whole slide image.

**Figure 6:**
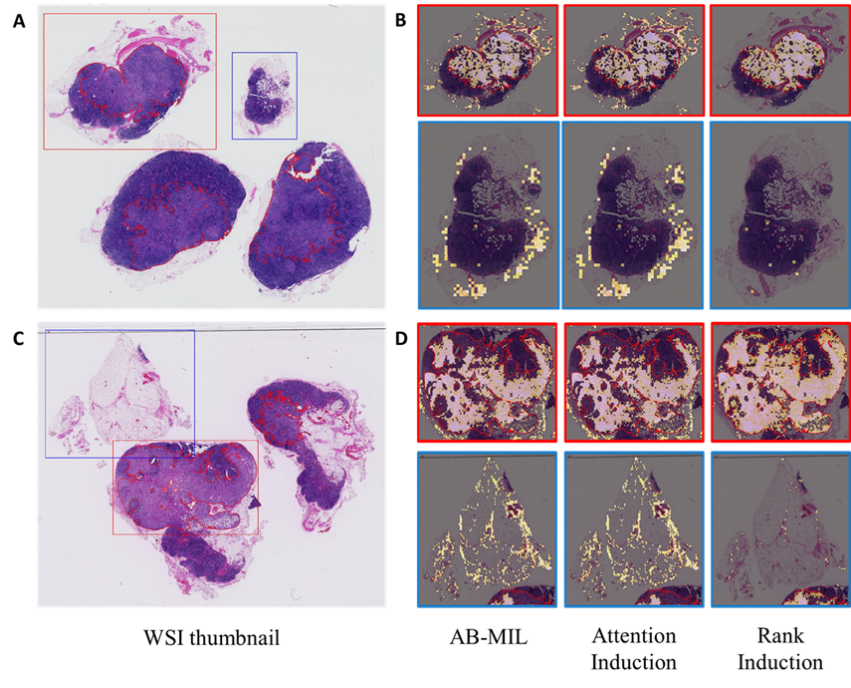
Qualitative visualization of patch selection for attention-based multiple instance learning (AB-MIL), attention induction, and rank induction. (A) A positive WSI thumbnail. (B) Attention distributions from each model in the lesion (red box) and nonlesion (blue box) regions in (A). (C) Another positive WSI thumbnail. (D) Attention distributions from each model in the lesion and nonlesion regions in (C).

To evaluate the effect of rank induction on attention localization, we examined whether the attention weights were concentrated within expert-annotated regions. Across all 3 localization metrics, rank induction consistently demonstrated greater focus on the annotated areas compared to other MIL methods. At α=5, AB-MIL, attention induction, and rank induction achieved IoU scores of 0.029, 0.023, and 0.031 and Dice scores of 0.056, 0.045, and 0.059, respectively. When α increased to 75, the performance gap between rank induction and the baseline models became more pronounced (Figures 5A and 5B). A similar trend was observed in pointing game accuracy (Figure 5C). Qualitatively, both AB-MIL and attention induction produced noisy attention maps that highlighted not only tumor regions but also large areas of normal tissue, resulting in frequent false positives. In contrast, rank induction generated sharp and well-localized attention maps, accurately delineating lesion areas with minimal off-target activation—closely matching the ground truth annotations. This distinct separation between tumor and normal regions explains rank induction’s superior performance and enhances the interpretability of its predictions (Figure 6).

## Discussion

### Principal Findings

In this work, we present rank induction, the first method to integrate MIL with expert annotations using pairwise rank constraints. By enforcing a ranking loss that encourages higher attention scores for annotated patches than for nonannotated ones, our approach effectively strengthens the weak supervision signal inherent to MIL frameworks.

We validated rank induction through comprehensive experiments on 2 public datasets and 1 in-house dataset, covering 5 key scenarios: full-data classification, low-data regimes, varying annotation granularity, subset annotation settings, and qualitative visualization. Across all settings, rank induction consistently outperformed baseline methods, including pseudo-labeling approaches and attention induction, while producing more faithful and interpretable attention heat maps.

Notably, the proposed ranking mechanism is model-agnostic and can be used as a versatile module within other MIL architectures. As demonstrated in Multimedia Appendix 3, integrating rank induction into existing methods such as DS-MIL and HIPT consistently improved their performance. In our main experiments, these baseline models (DS-MIL and HIPT) exhibited lower performance than that reported in their original studies. This discrepancy arises because we used a standard ImageNet-pretrained ResNet-50 backbone across all models to ensure a fair comparison of the MIL aggregation strategies themselves, rather than relying on the self-supervised pretraining typically required by those methods. However, even under this constrained setting, incorporating rank induction significantly boosted their classification accuracy, demonstrating the robustness and generalizability of our ranking-based supervision.

Ultimately, these performance improvements stem from bridging weak and strong supervision through informative pairwise constraints that approximate expert guidance. Rank induction is grounded in a simple yet clinically intuitive assumption: models should prioritize annotated regions over nonannotated ones. This inductive bias aligns naturally with domain knowledge in pathology, establishing rank induction as an effective and practical solution for real-world applications.

### Real-World Application With Expert Annotations

The pairwise ranking approach in rank induction addresses key challenges associated with expert annotations in real-world settings. In practice, tumor regions often exhibit irregular and heterogeneous morphology with poorly defined boundaries, making precise annotations difficult. Consequently, annotations are frequently coarse or incomplete. Despite this, rank induction maintained robust classification performance even with noisy annotations (Figure 3). This resilience likely stems from the pairwise training mechanism: although some comparisons may involve incorrectly labeled patches, most pairwise relationships remain valid, enabling the model to suppress noise and learn effectively.

Moreover, due to time and resource limitations, only a subset of positive WSIs can typically be annotated in clinical workflows. In such scenarios, extracting maximum value from limited expert input is critical. Unlike attention induction, which relies on assigning exact attention weights to individual patches, rank induction leverages a larger number of patch-pair comparisons from the valid pair set *P*, which grows quadratically with the number of annotated patches. This design allows the model to achieve near-saturated performance even with partial annotations (Figure 4).

Although pseudolabeling offers an alternative when expert annotations are unavailable, as seen in methods such as CLAM, it introduces uncertainty by relying on the model’s own predictions. These pseudolabels are inherently noisier than expert supervision and can lead to cascading errors. In contrast, rank induction avoids this risk by grounding supervision in verified expert annotations, leading to more stable and accurate slide-level predictions, especially in low-data or noisy-label conditions.

### Comparison of Fully Supervised Learning and Rank Induction

In scenarios where exhaustive patch-level annotations are available for all WSIs, fully supervised learning remains a strong baseline. Using the same training settings as our MIL models, a patch-level convolutional neural network with a fine-tuned ResNet-50 backbone and max-pooling aggregation achieved an AUROC of 0.892 on Camelyon16. This result outperformed the AUROC of 0.839 achieved by rank induction, which utilizes a frozen backbone and MIL-based aggregation. This result illustrates the upper bound of fully supervised performance given complete expert annotations and domain-specific fine-tuning (Multimedia Appendix 4).

However, in realistic clinical environments, exhaustive annotation is rarely feasible. When only 10% (9/89) of positive WSIs were annotated in the train dataset, the fully supervised model trained on this subset achieved an AUROC of 0.729. In contrast, rank induction, using the same 10% of patch-level annotations and leveraging slide-level labels from the remaining 90%, achieved a higher AUROC of 0.775. The key difference lies in how each method handles unannotated slides. Fully supervised models are limited to the annotated subset and cannot benefit from weak labels. Rank induction, by integrating both patch- and slide-level supervision through pairwise ranking constraints, makes effective use of all available data, even with sparse annotations.

These findings highlight a clear trade-off: while fully supervised learning performs best under ideal, fully annotated conditions, rank induction offers a more practical and annotation-efficient alternative. Its ability to generalize from partial supervision makes it especially suitable for real-world digital pathology applications, where annotations are incomplete or expensive.

### Limitations

Our study has several limitations. First, although we validated our method on 3 datasets, including 1 in-house dataset (SMF-stomach), the diversity of organs and pathologies remains limited. Specifically, the SMF-stomach dataset was collected from a single institution, which may introduce biases related to specific staining protocols or scanner characteristics. To fully assess cross-domain robustness, future work should include evaluation on larger, multicenter cohorts with diverse scanners. Nonetheless, the inclusion of Camelyon16 partially addresses this concern, as it contains slides from multiple medical institutions, providing evidence of generalization.

### Conclusions

In summary, rank induction provides a practical and effective solution for improving slide-level classification in digital pathology by bridging weak and strong supervision via pairwise ranking constraints. By leveraging only sparse expert annotations, it achieves a strong balance between annotation efficiency and model performance, outperforming traditional MIL and pseudolabeling approaches, particularly in low-data or noisy-label settings.

Although fully supervised learning may yield higher accuracy when exhaustive annotations are available, rank induction offers a more scalable and annotation-efficient alternative for real-world use. Its robustness with limited annotations and its ability to highlight diagnostically relevant regions underscore the potential of rank-based supervision to improve both model interpretability and generalizability across diverse clinical environments.

## Appendix

Multimedia Appendix 1 Example of a partially annotated whole slide image from the Seegene Medical Foundation-stomach dataset.

Multimedia Appendix 2 Comparison of slide-level classification accuracy across different multiple instance learning methods on 3 datasets.

Multimedia Appendix 3 Impact of integrating rank induction into existing multiple instance learning architectures (dual-stream multiple instance learning and hierarchical image pyramid transformer).

Multimedia Appendix 4 Performance comparison between fully supervised learning and rank induction under varying proportions of annotated positive whole slide images.

## Abbreviations

AB-MIL: attention-based multiple instance learning
AUPRC: area under the precision-recall curve
AUROC: area under the receiver operating characteristic curve
CLAM: clustering-constrained attention multiple instance learning
CLAM-MB: clustering-constrained attention multiple instance learning, multibranch
CLAM-SB: clustering-constrained attention multiple instance learning, single-branch
DS-MIL: dual-stream multiple instance learning
HIPT: hierarchical image pyramid transformer
IoU: intersection over union
IRB: institutional review board
MIL: multiple instance learning
SMF-stomach: Seegene Medical Foundation-stomach
WSI: whole slide image
