# Supplementary figures and images for: Ranking-Aware Multiple Instance Learning for Histopathology Slide Classification: Development and Validation Study
Source: JMIR Med Inform. 2026 Feb 4;14:e84417. doi: 10.2196/84417 (PMC12917480; doi:10.2196/84417)

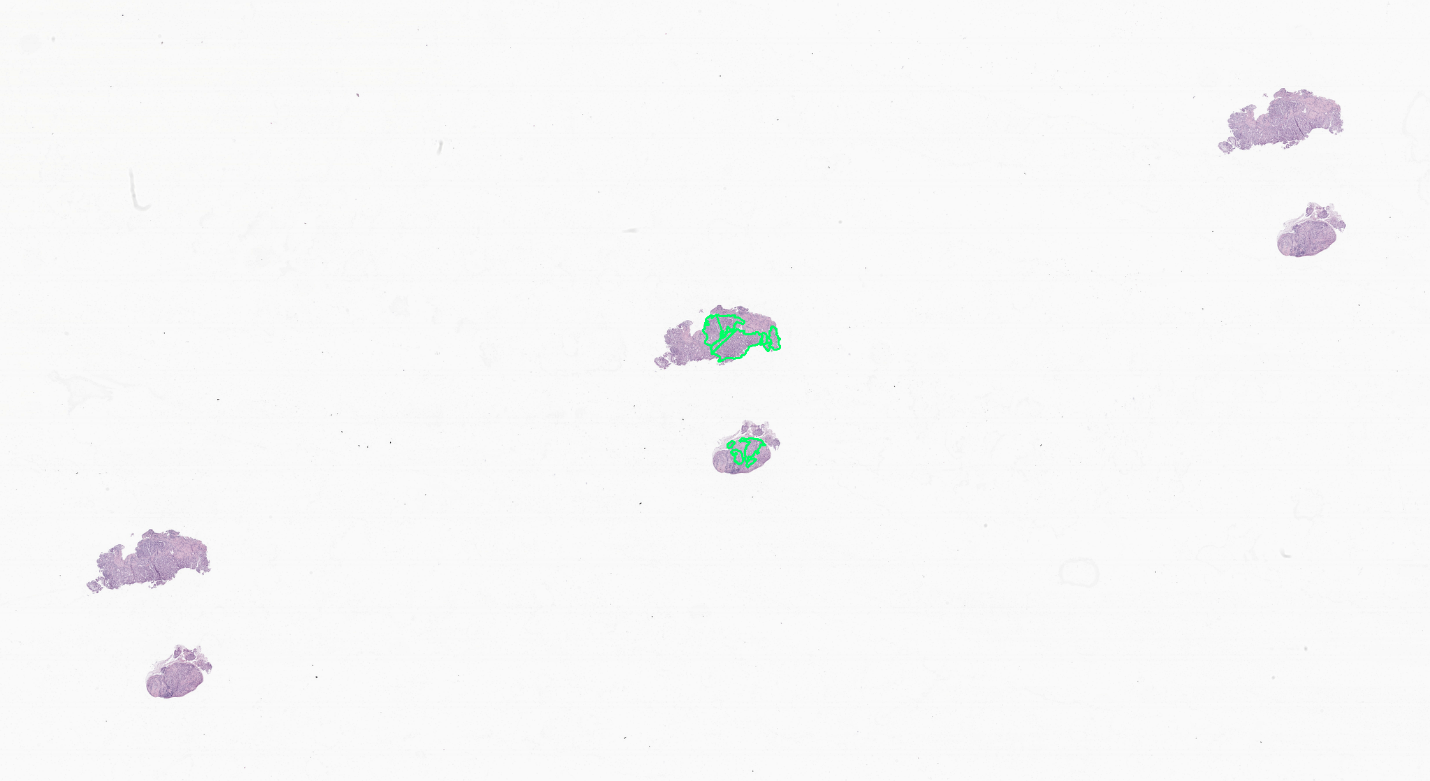

Supplement: Multimedia Appendix 1 [file medinform_v14i1e84417_app1.png]

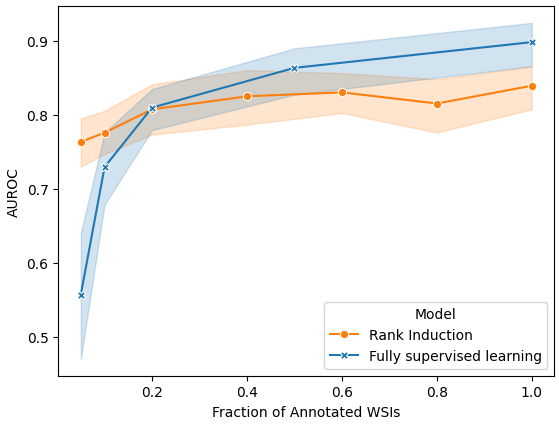

Supplement: Multimedia Appendix 4 [file medinform_v14i1e84417_app4.png]
