# Supplementary material for: Ranking-Aware Multiple Instance Learning for Histopathology Slide Classification: Development and Validation Study
Source: JMIR Med Inform. 2026 Feb 4;14:e84417. doi: 10.2196/84417 (PMC12917480; doi:10.2196/84417)
Supplement: Multimedia Appendix 2 [file medinform_v14i1e84417_app2.docx]

# **Multimedia appendix 2**

Supplementary Table 1. Comparison of slide-level classification accuracy across different MIL methods on three datasets

| Model | Camelyon16 | DigestPath2019 | SMF-stomach |
| --- | --- | --- | --- |
| AB-MIL | 0.778 ± 0.093 | 0.964 ± 0.017 | **0.778 ± 0.042** |
| Attention Induction | 0.765 ± 0.084 | **0.967 ± 0.013** | 0.771 ± 0.036 |
| CLAM-SB | 0.760 ± 0.087 | 0.910 ± 0.036 | 0.749 ± 0.031 |
| CLAM-MB | 0.783 ± 0.090 | 0.898 ± 0.041 | 0.754 ± 0.041 |
| DS-MIL | 0.740 ± 0.112 | 0.966 ± 0.013 | 0.732 ± 0.050 |
| HIPT | 0.638 ± 0.053 | 0.909 ± 0.086 | 0.675 ± 0.086 |
| Rank Induction | **0.838 ± 0.022**^abcef^ | 0.964 ± 0.015^cdf^ | 0.775 ± 0.037^cef^ |
| - Best result shown in bold, second-best underlined | | | |
| - Test accuracy was computed using a fixed slide-level decision threshold of 0.5 for all methods. | | | |
| - a: $P<.05$ vs AB-MIL; b: $P<.05$ vs Attention Induction; c: $P<.05$ vs CLAM-SB; d: $P<.05$ vs CLAM-MB; e: $P<.05$ vs DS-MIL; f: $P<.05$ vs HIPT | | | |
