# Supplementary material for: Ranking-Aware Multiple Instance Learning for Histopathology Slide Classification: Development and Validation Study
Source: JMIR Med Inform. 2026 Feb 4;14:e84417. doi: 10.2196/84417 (PMC12917480; doi:10.2196/84417)
Supplement: Multimedia Appendix 3 [file medinform_v14i1e84417_app3.docx]

# **Multimedia appendix 3**

Table. Impact of integrating Rank Induction into existing MIL architectures (DS-MIL and HIPT)

| Dataset | Model | Induction | AUROC | AURPC | Test ACC |
| --- | --- | --- | --- | --- | --- |
| Camelyon16 | DSMIL | Base | 0.690 ± 0.170 | 0.666 ± 0.223 | 0.740 ± 0.112 |
|  |  | Attention | 0.670 ± 0.163 | 0.652 ± 0.210 | 0.740 ± 0.099 |
|  |  | Rank | 0.760 ± 0.063 | 0.765 ± 0.055 | 0.778 ± 0.043 |
|  | HIPT | Base | 0.483 ± 0.103 | 0.416 ± 0.136 | 0.638 ± 0.053 |
|  |  | Attention | 0.484 ± 0.102 | 0.417 ± 0.115 | 0.629 ± 0.049 |
|  |  | Rank | 0.515 ± 0.119 | 0.454 ± 0.144 | 0.653 ± 0.064 |
| DigestPath2019 | DSMIL | Base | 0.995 ± 0.004 | 0.993 ± 0.006 | 0.966 ± 0.013 |
|  |  | Attention | 0.993 ± 0.005 | 0.991 ± 0.006 | 0.955 ± 0.021 |
|  |  | Rank | 0.996 ± 0.002 | 0.994 ± 0.003 | 0.971 ± 0.010 |
|  | HIPT | Base | 0.962 ± 0.058 | 0.947 ± 0.091 | 0.909 ± 0.086 |
|  |  | Attention | 0.889 ± 0.132 | 0.846 ± 0.181 | 0.841 ± 0.124 |
|  |  | Rank | 0.937 ± 0.064 | 0.897 ± 0.097 | 0.874 ± 0.077 |
| SMF-stomach | DSMIL | Base | 0.839 ± 0.031 | 0.915 ± 0.016 | 0.732 ± 0.050 |
|  |  | Attention | 0.843 ± 0.031 | 0.917 ± 0.016 | 0.744 ± 0.046 |
|  |  | Rank | 0.879 ± 0.015 | 0.939 ± 0.010 | 0.783 ± 0.036 |
|  | HIPT | Base | 0.676 ± 0.064 | 0.751 ± 0.060 | 0.675 ± 0.086 |
|  |  | Attention | 0.675 ± 0.042 | 0.744 ± 0.060 | 0.695 ± 0.032 |
|  |  | Rank | 0.717 ± 0.047 | 0.787 ± 0.052 | 0.714 ± 0.040 |
